# Pancreatic Stone Protein as an Early Predictor of Adverse Events in Patients with Infection Presenting to the Emergency Department: A Pilot Study

**DOI:** 10.3390/jpm16060312

**Published:** 2026-06-11

**Authors:** Louiza Mpoumi, Georgia Sarantos, Vasiliki Bistola, Sofia Bezati, Christos Verras, Ioanna Rita, Sotirios Tsiodras, John Parissis, Effie Polyzogopoulou

**Affiliations:** 1 Department of Emergency Medicine, Attikon University Hospital, National and Kapodistrian University of Athens, Rimini 1, 12462 Athens, Greece; louizampoumi@gmail.com (L.M.); georgiasarantou@yahoo.de (G.S.); vasobistola@yahoo.com (V.B.); sofiabezati@gmail.com (S.B.); christos.verras@gmail.com (C.V.); jane_rit@yahoo.gr (I.R.); jparissis@yahoo.com (J.P.); 2 4th Department of Internal Medicine, Attikon University Hospital, Rimini 1, 12462 Athens, Greece; sotirios.tsiodras@gmail.com

**Keywords:** infection, point-of-care, biomarker, pancreatic stone protein, emergency department

## Abstract

**Background**: Pancreatic stone protein (PSP) has recently emerged as a novel biomarker with diagnostic and prognostic potential in sepsis. The present study aimed to investigate its role as an early prognosticator in patients presenting to the Emergency Department (ED) with various types of infection. **Methods**: Point-of-care PSP was measured in 102 consecutive patients (59.8% male) with mean age of 62.7 (±23.4) years, presenting to the ED with suspected or confirmed infection. We examined the utility of PSP to predict adverse events including death, development of septic shock or need for repeated medical evaluation due to persistence or worsening of initial symptoms during a 10-day follow-up period. **Results**: Respiratory tract infections were the most common (50%) followed by urinary tract infections (17.6%), sepsis of unknown origin (4.9%) and other infections (27.5%). PSP exhibited intermediate performance in predicting short-term adverse outcomes with an AUC of 0.734 (*p* < 0.001). In contrast, other inflammatory biomarkers such as procalcitonin, C-reactive protein (CRP) and White Blood Cells (WBCs) did not predict adverse outcomes (procalcitonin: AUC 0.680, *p* = 0.059; CRP: AUC 0.593, *p* = 0.072; WBC: AUC 0.635, *p* = 0.074). **Conclusions**: PSP appears to be a promising biomarker reflecting the severity of infection. Point-of-care PSP evaluation may serve as an early predictor of adverse events in patients presenting with infection to the ED.

## 1. Introduction

Bacterial infections of various origins are among the most common causes for Emergency Department (ED) visits and are associated with a wide range of clinical outcomes, from mild illness to severe complications, including septic shock, hospitalization, and death. Early identification of patients at risk of clinical deterioration should be timely and safe to ensure appropriate management [1,2].

Ιn addition to clinical examination, classic biomarkers such as White Blood Cells (WBCs), C-reactive protein (CRP), lactic acid, procalcitonin (PCT) and scoring systems, including quick Sequential Organ Failure Assessment (qSOFA) [3], and National Early Warning Score 2 (NEWS2) [4] are useful for the identification and classification of severity in patients with infection, guiding treatment options [2,5,6,7]. However, these biomarkers provide limited reliability regarding the prognosis of adverse events [2,5]. As a result, there is an increasing demand for the development of novel biomarkers that may enhance identification of patients with severe infections at presentation but also discriminate those at risk of further clinical deterioration [8,9]. In this context, these biomarkers could also play a role in determining whether a patient can be safely discharged from the ED.

Pancreatic stone protein (PSP) has emerged as a novel biomarker of inflammation, reflecting endogenous stress [10,11]. Compared to traditional inflammatory biomarkers, PSP increases early in response to systemic stress and infection. It was historically discovered in 1980 as a protein released in conditions of pancreatic damage, known as regenerating protein 1α (REG1A). Further research showed its multifactorial role as an acute phase protein in conditions of systemic inflammation and infection [12]. Its utility as a biomarker is attributed to its cardinal role in the pathophysiology of systemic inflammation [13], as it contributes to the activation of neutrophils and the enhancement of immunity.

A key advantage is that PSP can also be measured rapidly using point-of-care (POC) systems, facilitating prompt clinical decision-making in the ED. Several studies in Intensive Care Unit (ICU) have reported correlation between increased serial PSP concentrations and greater disease severity or mortality [10,11]. Recently, PSP has been studied in patients presenting to the ED with infection in order to identify its prognostic potential [14,15]. However, most of these studies are focused on overt sepsis [16,17,18] while the data for PSP in patients with infections of heterogeneous origin remain limited [14,15,18] in the emergency setting. The aim of this study is to evaluate the prognostic value of bedside PSP measured upon presentation to the ED for the prediction of adverse clinical events in patients presenting with a wide range of infection severity.

## 2. Materials and Methods

### 2.1. Study Population

This is a prospective observational study conducted in the ED of Attikon University Hospital, Athens, Greece, a tertiary University hospital from May to August 2025. We enrolled patients presenting with clinical features suggestive of infection including lower respiratory tract infection, urinary tract infection, intra-abdominal infection, soft tissue infection, infection of central nervous system and sepsis of unknown origin. More specifically, suspected infections were defined based on the presence of clinical signs and symptoms related to the suspected site of infection at the time of ED evaluation. Clinical manifestations included systemic features such as fever, fatigue, malaise, nausea, and organ-specific symptoms such as new onset of cough/purulent sputum, dyspnea, sore throat for respiratory tract infections, dysouria and/or flank pain for urinary tract infections, abdominal pain, vomiting or diarrhea for intra-abdominal infections, erythema, warmth and/or swelling for soft tissue infection and headache, photophobia or altered mental status for central nervous system infections. Sepsis was defined according to the Surviving Sepsis Campaign guidelines [19]. Exclusion criteria were age < 18 years, immunosuppression due to neutropenia (<1.000 neutrophils/μL) or severe hematological disease, acute or chronic pancreatitis, prior pancreatic surgery, pancreatic cancer or other gastrointestinal malignancy, end-stage renal disease or hemodialysis and pregnancy. All patients or their next of kin provided written informed consent before enrollment. The study was conducted in accordance with the Declaration of Helsinki and was approved by the institutional review board (IRB) of Attikon University Hospital (Approval number 327-05/05/2025).

### 2.2. Study Procedures

Upon patient presentation, baseline characteristics such as age, sex and comorbidities were collected. Vital signs including respiratory rate, blood oxygen saturation, blood pressure, heart rate and body temperature were recorded. Patients with infection were triaged with respect to the severity of symptoms according to the Emergency Severity Index (ESI) score [20]. Moreover, we calculated the quick Sepsis-Related Organ Failure Assessment (qSOFA) [3] and National Early Warning Score 2 (NEWS 2) [4] prognostic scores.

During initial evaluation, blood samples were collected for blood gas analysis, complete blood count, including White Blood Cells (WBCs), basic biochemistry panel, including creatinine, and biomarkers of inflammation, namely C-reactive protein (CRP), procalcitonin (PCT), and soluble urokinase Plasminogen Activator Receptor (SUPAR). All routine laboratory testing was performed in real time at the hospital central laboratory. In addition, a whole blood sample was obtained upon ED presentation for the measurement of PSP at the patient bedside using a point-of-care device (abioSCOPE, Abionic company, Epalinges, Switzerland), via the fluorescence immunoassay method that provides quantitative results within 5 min. The abioSCOPE uses a single-use diagnostic capsule in vitro, in venous or capillary whole blood, mixed with K3-EDTA or K2-EDTA anticoagulant. Blood cultures were also collected in the presence of clinical indication and according to the Surviving Sepsis Campaign guidelines [19]. PSP measurements were available only to the investigator and not to the treating physicians; therefore, PSP values had no influence on the diagnostic assessment, therapeutic management, or disposition decisions of the patients throughout the study period.

Upon completion of ED evaluation, patients were either discharged or admitted to the hospital for further management based on the local protocol and clinical judgment of the attending physician. Follow-up information regarding the occurrence of adverse clinical events was obtained from the hospital-based patient registry and through phone interviews.

### 2.3. Outcomes

The primary endpoint was the occurrence of any adverse clinical event within 10 days after ED presentation. Adverse clinical events were defined as severe, including all-cause mortality and development of septic shock, or minor, defined by the need for ED revisit due to worsening symptoms of the primary illness. Specifically, ED revisits were included only when patients returned due to persistent or worsening symptoms of the original infectious episode that had not resolved after ED discharge. Revisits for unrelated medical complaints were not considered study endpoints.

### 2.4. Statistical Analysis

Statistical analysis was performed using SPSS version 29.0 (SPSS, Inc., Chicago, IL, USA). Categorical variables are presented as frequencies and percentages, whereas quantitative variables are expressed as means with standard deviations (SDs) or as medians with interquartile ranges (IQRs) for normally or non-normally distributed variables, as indicated by the Kolmogorov–Smirnov test. Differences between subgroups of patients who had an adverse clinical event and ones without adverse clinical events were tested using chi-square test for qualitative parameters and Mann–Whitney test for quantitative parameters. Spearman’s correlation coefficients were used to investigate correlations between non-normally distributed continuous variables. The values of laboratory variables were log-transformed for regression analysis due to their highly skewed distribution. In order to examine the association between PSP levels upon ED presentation and outcomes, receiver operator characteristic (ROC) analysis and univariate logistic regression were performed. A secondary analysis was also performed to explore the association between PSP levels and severe adverse outcomes. Head to head comparison of biomarkers regarding their ability to predict the primary and secondary end-points was performed using ROC curve data analysis (*n* = 91). Post hoc power calculation showed that the study had 72.1% power to detect the observed difference in PSP levels between patients with and without adverse events within 10 days after ED presentation, at an alpha level of 0.05. A *p* value of <0.05 was considered to be statistically significant.

## 3. Results

### 3.1. Baseline Characteristics

In this study, 102 patients were enrolled with a mean age of 62.7 (±23) years and 59.8% were males. Baseline characteristics of the total population are described in Table 1. A total of 75 patients (73.5%) were admitted to the internal medicine ward and 27 patients (26.5%) were discharged upon first ED evaluation. In the entire cohort, during the 10-day follow-up, 19 patients (18.6%) had an adverse event; 12 died, 6 deteriorated to sepsis/septic shock and 1 needed hospitalization for the same infection after ED discharge.

Respiratory infections were the most common and occurred in 51 patients (50%), followed by intra-abdominal infections diagnosed in 19 patients (18.6%), urinary tract infections in 18 patients (17.6%), sepsis of unknown etiology in 5 patients (4.9%) and other infections in 9 patients (8.8%), of whom 3 (2.9%) had central nervous system infection and 6 (5.9%) had soft tissue infection. Among patients with intra-abdominal infections, 7 (6.9%) had cholecystitis, 5 (4.9%) cholangitis, 3 (2.9%) appendicitis and 4 (3.9%) infection caused by *Clostridioides difficile*.

Patients who experienced adverse events were more frequently older and had higher prevalence of dementia, atrial fibrillation and malignancy. Patients who experienced adverse events presented with significantly higher levels of PSP compared to patients without adverse events [198 ng/mL (74–384) vs. 73 ng/mL (30–188.5) (*p* = 0.004), respectively]. Moreover, they had significantly higher levels of PCT (*p* = 0.018), lactic acid (*p* = 0.043), creatinine (*p* = 0.001), and suPAR (*p* = 0.003). Interestingly, they were more frequently classified with high-acuity ESI score [15.8% and 31.6% were classified as ESI 1 or 2, respectively (*p* < 0.006)] and presented with a higher qSOFA [73.7% had a qSOFA ≥ 2, (*p* < 0.001)] and NEWS 2 score [68.4% classified as high risk (>7) (*p* < 0.001)].

Blood cultures were obtained in 38 patients (37.2%), of whom 10 were positive for a bacterial agent. PSP levels were significantly higher in patients with bacteremia compared to patients who had a negative blood culture [327 ng/mL (198.75–446.7) vs. 125.5 ng/mL (58–227.2) (*p* = 0.021), respectively]. Levels of other biomarkers of inflammation did not differ significantly in patients with positive blood culture vs. patients with negative blood culture; CRP (*p* = 0.486), PCT (*p* = 0.644), WBC (*p* = 0.380) and suPAR (*p* = 0.354). Details of the laboratory analyses of patients with blood culture obtained are described in Table 2.

### 3.2. Correlations

Spearman’s correlation analysis revealed significant positive correlations between PSP levels and other biomarkers of inflammation. The correlation was strong with suPAR levels (rho = 0.603, *p* < 0.001) and moderate with procalcitonin levels (rho = 0.505, *p* < 0.001). Further analysis revealed a weak positive correlation between PSP levels and WBC s (rho = 0.280, *p* = 0.004) or lactate (rho = 0.279, *p* = 0.005). Interestingly, Spearman’s correlation showed no significant relationship between PSP and CRP levels (*p* > 0.05).

### 3.3. Prognostic Role of PSP

**Figure 1:**
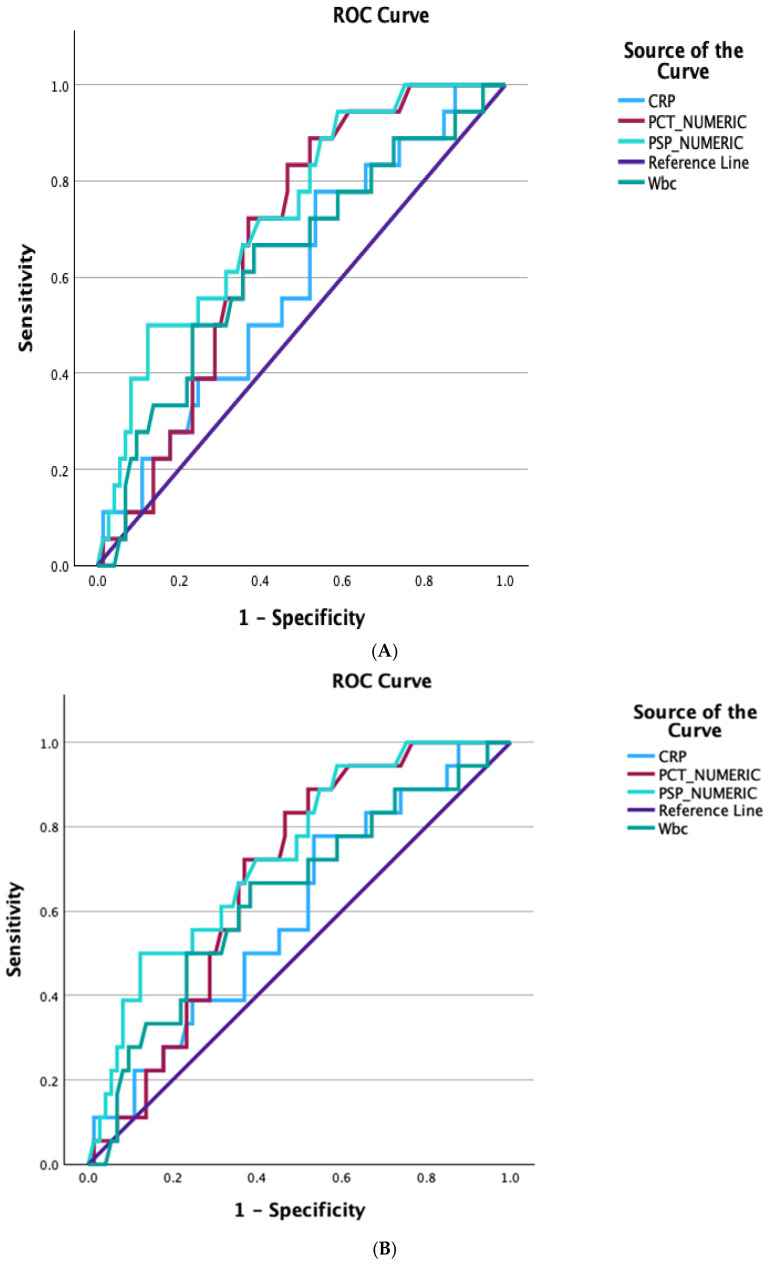
(**A**). Head to head comparison of Receiver operating characteristic (ROC) curves of C-reactive protein (CRP), procalcitonin (PCT), pancreatic stone protein (PSP) and White blood cells (Wbcs) for predicting primary outcome. (**B**). Head to head comparison of Receiver operating characteristic (ROC) curves of C-reactive protein (CRP), procalcitonin (PCT), pancreatic stone protein (PSP) and White blood cells (Wbcs) for predicting secondary outcome.

Using regression analysis, logPSP was associated with the occurrence of adverse events (death, septic shock or ED revisit), [odds ratio (OR), 95% confidence interval (CI): 5.887, 1.68–20.59, *p* = 0.06]. In a secondary outcome analysis, including only severe adverse events (death or progression to septic shock), logPSP was associated with the occurrence of severe adverse events, as well, [odds ratio (OR), 95% confidence interval (CI): 6.707, 1.82–24.69, *p* = 0.004]. Head to head comparison of predictive ability of biomarkers for the primary (Figure 1A) and secondary outcomes (Figure 1B) showed that PSP demonstrated an intermediate predictive ability for both outcomes (primary outcome: AUC 0.734, 95% CI: 0.613–0.856, *p* < 0.001; secondary outcome: AUC 0.750, 95% CI: 0.629–0.872, *p* < 0.001), followed by PCT (primary outcome: AUC 0.680, 95% CI: 0.564–0.796, *p* = 0.059; secondary outcome: AUC 0.709, 95% CI: 0.601–0.818, *p* = 0.055), WBC (primary outcome: AUC 0.635, 95% CI: 0.490–0.781, *p* = 0.074; secondary outcome: AUC 0.650, 95% CI: 0.500–0.800, *p* = 0.077), and CRP (primary outcome: AUC 0.593, 95% CI: 0.452–0.734, *p* = 0.072; secondary outcome: AUC 0.595, 95% CI: 0.448–0.742, *p* = 0.075).

A PSP cut-off value of 66 ng/mL could predict primary outcome adverse events with a sensitivity of 88.9% and a specificity of 45.2%.

## 4. Discussion

In this prospective observational pilot study, we examined the predictive ability of POC PSP levels for short-term adverse events in patients presenting with infection in the ED. Firstly, we demonstrated that PSP levels were significantly higher in patients who developed short-term adverse events, compared to patients with no adverse events documented. Moreover, PSP levels were significantly higher in patients with bacteremia, suggesting its upregulation according to the severity of infection in the early stages of the disease course. Our observation is consistent with previous reports suggesting that PSP elevation may precede clinical manifestations of sepsis for up to 72 h [17,18]. Interestingly, its increase is time-dependent, reflecting the severity of infection in relation to systemic stress at the time its levels are measured, suggesting its usefulness as an early biomarker in the acute care setting [21]. Recent literature suggests that it is upregulated in sepsis due to tissue destruction and systemic stress and correlates with the severity of organ dysfunction [11,22].

Additionally, baseline PSP levels were independently associated with risk for increased short-term morbidity and mortality and showed moderate prognostic accuracy for the occurrence of adverse events, with an AUC of 0.75. Our findings are in line with other studies that reported the value of PSP as an early predictor of adverse outcomes [23,24] and mortality [16] in patients with undifferentiated infection. This is of particular importance, as it implies that PSP may serve as an adjunct rule-out tool, possibly embedded in a risk stratification score in everyday clinical practice. According to our analysis, a cut-off value of 66 ng/mL could detect patients with infection who are at low risk for further deterioration. Yet, further large scale studies are required in order to define a proper cut-off value, as proposed cut-offs in the literature present high variability; 48.5 ng/mL [23]–306 ng/mL [24].

Remarkably, in this study, PSP levels demonstrated higher predictive ability compared to other established inflammatory biomarkers such as WBCs and CRP. WBCs showed a modest discriminatory ability for severe adverse events and CRP showed no statistically significant results regarding the occurrence of adverse events. While the use of CRP and WBCs is widespread and they are more routinely used in patients with infection, they are less time-dependent and less specific regarding the identification and the severity of infection [25,26]. Our findings may reflect the more generic profile of these parameters and suggest their inability to serve as prognostic tools for adverse outcomes. A similar pattern is seen with another biomarker, suPAR, which although it has been proposed for safe discharge of patients with infection, it remains out of routine clinical practice for decision-making in the ED [27]. Moreover, procalcitonin showed moderate prognostic utility, slightly inferior to PSP. Indeed, according to the latest evidence, it seems to subside as a diagnostic tool and is currently recommended mainly for guiding antibiotic de-escalation, according to sepsis guidelines [19].

In the ED, despite the ongoing development of novel biomarkers, there are still no widely accepted and established biomarkers that provide early recognition of patients with moderate disease severity and subtle clinical presentation who may subsequently deteriorate. PSP has the potential to serve as a useful risk stratification tool for the management of patients with undifferentiated infection in the acute setting.

Our study has some important strengths. Firstly, we studied patients presenting to the ED and we used a POC device. Previous literature on the role of PSP focused on patients in the ICU setting, while studies in the ED are limited. With the advent of a point-of-care device and the establishing role of PSP as a diagnostic and prognostic tool, several study groups have focused on the role of PSP in the ED [18,22,23,24]. Measurement of PSP with a POC device offers the advantage of early risk stratification and management of patients with infection. Yet, further research is needed to validate its utility so as to establish a definite cut-off value aiding in the triage and disposition of patients with infection. Secondly, we included patients with infections of various etiologies, aiming to contribute to the limited body of literature in this field, as many studies have focused on specific sites of infection, such as intra-abdominal infections [28]. Therefore, we provide evidence for the prognostic utility of PSP in a wider spectrum of infections [14,15]. In addition, in accordance with the study of Michailides et al. who explored the predictive utility of PSP for sepsis and all-cause mortality [23], we did not only include hospitalized patients, but we also investigated the need for re-evaluation after ED discharge, aiming to examine the prognostic value of PSP in a real-world setting, including patients initially considered as low risk, who may deteriorate. Our study aimed to contribute to this gap of literature and provide additional information regarding the management and disposition of undifferentiated patients with infection. The scope of this pilot study was not to establish definitive clinical applicability, but rather to provide preliminary data that could support and stimulate further research in this direction.

Future research should focus on investigating the potential value of PSP in identifying patients with subtle clinical presentation and moderate disease severity (e.g., ESI level 3–4), who may be at increased risk of clinical deterioration. Additional studies could focus on its role as a biomarker for the early rule-out of patients at low risk for deterioration in the emergency setting. Furthermore, integrating PSP into existing clinical scores, such as qSOFA or NEWS2, or incorporating it into multi-biomarker models, may further enhance risk stratification and decision-making in the Emergency Department [29].

The main limitation of the study is that it is a single-center study, with a relatively small number of patients. Moreover, part of the study design was the delineation of exclusion criteria with the rationale to minimize potentially confounding conditions that could affect PSP levels. We excluded patients with gastrointestinal malignancies, immunosuppression related to neutropenia, or severe hematological disease. We also excluded patients with acute or chronic pancreatitis, severe renal failure and pancreatic cancer, as PSP has an absolute contraindication in such patients, according to the manufacturer’s recommendations. The small sample size and the populations excluded may limit generalizability of our results. Furthermore, the small sample size has restricted us from performing additional investigations regarding multivariate analysis. Therefore, further large-scale studies are needed to examine the importance of other variables as prognostic tools in patients with infection, make comparisons and explore the role of PSP in special pathologic conditions and populations.

## 5. Conclusions

PSP demonstrated a moderate predictive ability for the occurrence of severe adverse events in patients with infection of various origins presenting to the ED. Therefore, PSP may potentially reflect the severity of infection and could serve as a hypothesis-generating biomarker demanding additional investigation in order to further clarify the predictive performance, optimal clinical role, and potential integration of PSP clinical practice.

Point-of-care PSP evaluation may aid in the early risk stratification of patients presenting with infection to the ED. Further research is needed in order to validate these preliminary results and elucidate its prognostic role and its clinical utility in patients presenting with infection.

## Figures and Tables

**Table 1:** Baseline characteristics study population.

Study Population	All Patients	No Adverse Events	Adverse Events	*p*-Value
**N**	**102**	**83**	**19**	
**Demographics**				
**Male sex, n (%)**	61 (59.8)	48 (57.8)	13 (68.4)	0.396
**Age (years ± SD)**	62.7 (±23.4)	59.3 (±23.1)	76.4 (±20.4)	**0.003**
**Comorbidities, n (%)**				
Arterial Hypertension	38 (37.3)	29 (34.9)	9 (47.4)	0.350
Diabetes Mellitus	22 (21.6)	16 (19.3)	6 (31.6)	0.263
Dyslipidemia	20 (19.6)	15 (18.1)	5 (26.3)	0.444
Coronary Artery Disease	16 (15.7)	12 (14.5)	4 (21)	0.504
Dementia	15 (14.7)	9 (10.8)	6 (31.6)	**0.025**
Atrial Fibrillation	11 (10.8)	6 (7.2)	5 (26.3)	**0.018**
Chronic Obstructive Pulmonary Disease	11 (10.8)	10 (12)	1 (5.3)	0.375
Chronic Kidney Disease	7 (6.9)	4 (4.8)	3 (15.8)	0.095
Heart Failure	6 (5.9)	5 (6)	1 (5.3)	0.881
Cancer	5 (4.9)	2 (2.4)	3 (15.8)	**0.017**
**Clinical presentation**				
**Type of infection, n (%)**				**0.002**
Respiratory Infection	51 (50)	39 (47)	10 (52.6)	
Urinary Infection	18 (17.6)	17 (20.5)	1 (5.3)	
Sepsis of Unknown Origin	5 (4.9)	1 (1.2)	4 (21.1)	
Other	28 (27.5)	24 (28.9)	4 (21.1)	
**Laboratory analysis**				
**WBC** (cells/µL)	10.690 (7.860–15.280)	10.450 (7.450–14.565)	13.700 (8.480–19.300)	0.101
**CRP** (mg/L)	76.5 (30.1–184)	79 (28–184)	80.8 (46.5–203)	0.544
**PCT** (ng/mL)	0.17 (0.06–0.88)	0.13 (0.06–0.67)	0.45 (0.16–1.40)	**0.018**
**Lactic acid** (mmol/L)	1.5 (1.1–2.1)	1.4 (1.1–2.0)	1.7 (1.4–3.7)	**0.043**
**Creatinine** (mg/dL)	0.89 (0.74–1.4)	0.86 (0.69–1.09)	1.52 (1.0–2.01)	**0.001**
**suPAR (ng/mL)**	6.75 (4.7–13.2)	6.15 (4.2–9.7)	16.1 (8.15–34.75)	**0.003**
**PSP** (ng/mL)	93 (34–214)	73 (30–188.5)	198 (74–384)	**0.004**
**Risk scores**				
**NEWS 2, n (%)**				**<0.001**
Low (1–4)	57 (55.9)	53 (64.2)	3 (15.8)	
Low-Medium (+3)	9 (8.8)	8 (9.9)	1 (5.3)	
Medium (5–6)	10 (9.8)	8 (9.9)	2 (10.5)	
High (>7)	26 (25.5)	13 (16)	13 (68.4)	
**qSOFA** **, n (%)**				**<0.001**
Not high risk (0–1)	73 (71.6)	68 (81.9)	5 (26.3)	
High risk (≥2)	27 (26.5)	13 (15.7)	14 (73.7)	
Missing	2 (1.9)	2 (2.4)	0 (0)	
**ESI, n (%)**				**0.006**
1	10 (9.8)	7 (8.4)	3 (15.8)	
2	15 (14.7)	9 (10.8)	6 (31.6)	
3	25 (24.5)	23 (27.7)	2 (10.5)	
4	19 (18.6)	18 (21.7)	1 (5.3)	
5	0 (0)	0 (0)	0 (0)	
Missing	33 (32.4)	26 (31.3)	7 (36.8)	

Data are shown as n (%) for categorical variables, mean (SD) for continuous normally distributed variables or median (IQR) for continuous non-normally distributed variables. CRP, C-reactive protein; ESI, Emergency Severity Index; NEWS 2, National Early Warning Score 2; procalcitonin (PCT); PSP, Pancreatic Stone Protein; qSOFA, quick Sepsis-Related Organ Failure Assessment; suPAR, soluble urokinase Plasminogen Activator Receptor; WBC, White Blood Cells; bold for the statistically significant numbers.

**Table 2:** Laboratory parameters in the subgroup of patients with blood cultures obtained.

Patients with Blood Culture N = 38	Positive Blood Culture N = 28	Negative Blood Culture N = 10	*p* Value
Laboratory analysis			
PSP (ng/mL)	327 (198.75–446.7)	125.5 (58–227.2)	**0.021**
CRP (mg/L)	119.9 (50–251.7)	93.49 (11.55–185.5)	0.486
PCT (ng/mL)	0.83 (0.34–9.94)	0.54 (0.086–2.6)	0.644
WBC (cell/μL)	11.950 (8.112–23.072)	10.525 (8.145–13.872)	0.380
suPAR (ng/mL)	11.1 (6.32–38.3)	8.5 (5.55–15.95)	0.354

Data are shown as median (IQR) for all variables. CRP, C-reactive protein; procalcitonin (PCT); PSP, Pancreatic Stone Protein; suPAR, soluble urokinase Plasminogen Activator Receptor; WBC, White Blood Cells.

## Data Availability

The data are available from the corresponding author upon reasonable request.
